# The neural basis of human tool use

**DOI:** 10.3389/fpsyg.2014.00310

**Published:** 2014-04-09

**Authors:** Guy A. Orban, Fausto Caruana

**Affiliations:** ^1^Department of Neuroscience, University of ParmaParma, Italy; ^2^Brain Center for Social and Motor Cognition, Italian Institute of TechnologyParma, Italy

**Keywords:** tool use, affordances, mechanical problem solving, anterior supramarginal gyrus, anterior intraparietal sulcus

## Abstract

In this review, we propose that the neural basis for the spontaneous, diversified human tool use is an area devoted to the execution and observation of tool actions, located in the left anterior supramarginal gyrus (aSMG). The aSMG activation elicited by observing tool use is typical of human subjects, as macaques show no similar activation, even after an extensive training to use tools. The execution of tool actions, as well as their observation, requires the convergence upon aSMG of inputs from different parts of the dorsal and ventral visual streams. Non-semantic features of the target object may be provided by the posterior parietal cortex (PPC) for tool-object interaction, paralleling the well-known PPC input to anterior intraparietal (AIP) for hand-object interaction. Semantic information regarding tool identity, and knowledge of the typical manner of handling the tool, could be provided by inferior and middle regions of the temporal lobe. Somatosensory feedback and technical reasoning, as well as motor and intentional constraints also play roles during the planning of tool actions and consequently their signals likewise converge upon aSMG. We further propose that aSMG may have arisen though duplication of monkey AIP and invasion of the duplicate area by afferents from PPC providing distinct signals depending on the kinematics of the manipulative action. This duplication may have occurred when Homo Habilis or Homo Erectus emerged, generating the Oldowan or Acheulean Industrial complexes respectively. Hence tool use may have emerged during hominid evolution between bipedalism and language. We conclude that humans have two parietal systems involved in tool behavior: a biological circuit for grasping objects, including tools, and an artifactual system devoted specifically to tool use. Only the latter allows humans to understand the causal relationship between tool use and obtaining the goal, and is likely to be the basis of all technological developments.

## INTRODUCTION

The purpose of this short paper is to review the functional magnetic resonance imaging (fMRI) evidence related to the presence in the human brain of a region devoted to tool use lying in the anterior supramarginal gyrus (aSMG) of the left hemisphere, and to describe the properties of this area by integrating our findings with those of other imaging studies. Next, we derive the connections of this region active during the execution and observation of tool action and confront the cognitive operations implied by these results with views of the cognitive processes involved in tool use derived from neuropsychological studies of apraxia. Finally we discuss the emergence of tool use during evolution.

## THE DIFFERENCE BETWEEN HUMANS AND MONKEYS IN TOOL USE

Historically, tool use was considered a typically human behavior and the emergence of tool use was considered an important step in the evolution of primates, even serving to delineate the appearance of the genus Homo (Ambrose, 2001). It has, however, become increasingly clear that other animals, particularly chimpanzees and other old and new world monkeys do employ tools (for a review, van Schaik et al., 1999; Baber, 2003). Yet, even if actions using tools are simply compared between humans and apes, it becomes apparent that humans understand the causal relationship between the use of the tool and the results obtained, while this appears not to be the case for chimpanzees (Povinelli et al., 2000). It seems that in other species tool use can be understood by a combination of the affordances provided by the object, which can be manipulated in a tool-like fashion, and associative learning linking the presumptive tool and the result. As pointed out by Osiurak et al. (2010), the differences between animals and humans become even clearer if one makes the comparison both over the lifetime of an individual, in that humans use tools frequently and spontaneously, and at the species level, because all human societies develop technological devices which evolve and improve (Osiurak et al., 2010). Accordingly, it has been recently suggested that the striking differences between human and primates tool use reflect evolutionary discontinuities in hand-eye coordination, causal reasoning, function representation, executive control, social learning, teaching, social intelligence, and language (Vaesen, 2012), thus suggesting important differences with respect to brain structures and functions involved in tool use. The starting point of this review is that the study of the neural basis of tool use has made sufficient progress to begin to understand why primates such as monkeys can use tools and why the human tool use is so radically different from that of non-human primates.

More than 15 years ago, Iriki et al. (1996) observed that the body scheme could be modified by training macaques to use a rake to retrieve food that was otherwise out of reach. Bimodal neurons in the anterior intraparietal sulcus (IPS; presumably medial bank) having somatosensory receptive field (sRF) and visual receptive field (vRF) representing the finger tips expanded their vRF to include the entire tool after extensive tool use. A similar extension of the properties of the biological effector to the tool was observed in monkey ventral premotor cortex (vPMC) F5. Umiltà et al. (2008) showed that, after extensive training, hand grasping premotor neurons also become active during grasping with ordinary pliers and, more interestingly, with reverse pliers requiring finger extension, rather than flexion, to grasp the object. Such modifications of the neural apparatus involved in planning and controlling object manipulation are likely to underlie the capacity of macaques, and probably apes, to use simple tools, such as the twigs employed in fishing for termites. Some evidence has been obtained for similar changes in humans Maravita and Iriki (2004), and Rushworth et al. (2003) have suggested that such changes may underlie the responses to static tool images reported in left aSMG. Yet, these adaptations are unlikely to explain the causal understanding that humans have of tool use, or the extent of human tool use. In contrast, the sporadic use of tools in animals could be simply explained by such changes in the biological grasping circuit brought about by mere associative learning processes. Typically this animal behavior is based on using objects such as stones or twigs, readily available in the environment. This use may become conditioned by repeated success, including the choice of most appropriate objects. Indeed, in the above mentioned experiment of Umiltà et al. (2008), the use of complex pliers required an extensive training (6–8 months) and was actually based on associative learning, achieved in three subsequent, rewarded, steps: grasping the pliers, opening/closing the pliers and, finally, operating the pliers to grasp food.

Peeters et al. (2009) have more recently provided evidence for a neural substrate involved in tool use that is typically human. These authors compared observation of biological actions such as dragging or grasping, with observations of tool action having similar goals, in both humans and monkeys. They discovered that when human subjects observed tool actions a region in left aSMG was differentially active with respect to static controls, while the same region was not differentially active when subjects simply observed the biological actions, i.e., the factors *tool* and *action* interacted. This finding was very robust as it was observed in nearly 50 subjects, and tested with three different tool actions: using a screwdriver, a rake and pliers, as well as with actions performed by a robotic arm. Most interesting, when performing the same testing in monkeys, Peeters et al. (2009) failed to observe any similar interaction in the monkey inferior parietal lobule (IPL), even after extensive training when the animals had become proficient in using the rake and the pliers. These experiments indicated that the activation of left aSMG by tool action observations was a typically human trait. Since the same aSMG region has been reported to be activated in humans by pantomiming of tool use and the execution of tool actions (see below for references), Peeters et al. (2009) proposed that human, but not monkey cortex includes a region in the left supramarginal gyrus, devoted to executing and observing tool actions. It should be noted that these results do not exclude the possibility that in monkeys a few scattered neurons in the biological action observation circuit (Nelissen et al., 2011) respond to observation of tool actions. Even then, the results would still imply that in the human case the neurons responding to tool action observation are grouped together and therefore are computationally more powerful than the isolated neurons in the monkey. Indeed, the grouping of neurons with similar function is an important principle of cortical processing, as columnar and topographic organizations demonstrate. The clustering of face selective neurons in the face patches also illustrates the same principle (Tsao and Livingstone, 2008). Importantly, these findings of a left aSMG activation by tool action observation have been replicated in a new group of 12 subjects (Peeters et al., 2013), confirming the robustness of this finding.

In conclusion these studies allow us to understand how on one hand monkeys and apes can become efficient tool users, by modifying their biological manipulative action observation/execution circuit, and why humans use tools so extensively and so proficiently, by possessing a uniquely human cortical region devoted to the observation/execution of tool actions.

## THE LEFT aSMG REGION DEVOTED TO TOOL USE

**FIGURE 1:**
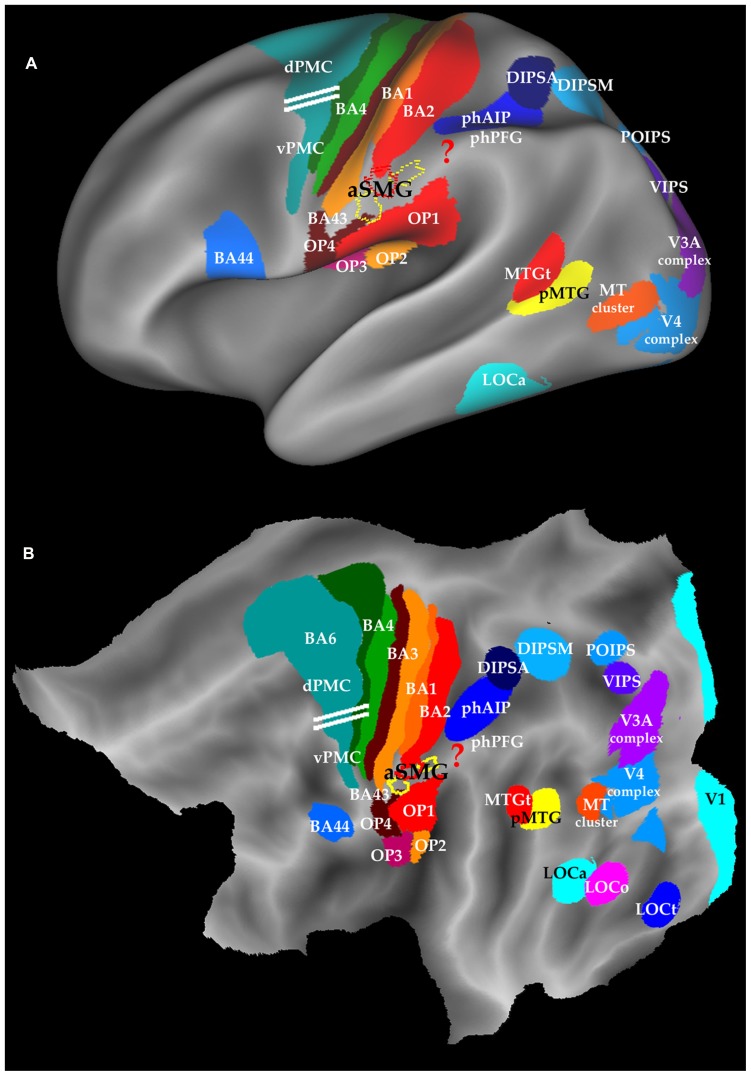
**Location of aSMG on the left inflated brain (A) and flatmap **(B)** of left hemisphere using Caret (Van Essen, 2005).** Yellow and red outlines: aSMG (Peeters et al., 2013); ? in red: area involved in technical reasoning; horizontal white lines : separation between dPMC and vPMC (Tomassini et al., 2007); BA: Brodmann area; dPMC and vPMC dorsal and ventral premotor cortex; OP: opercular areas, DIPSA, DIPSM, POIPS, VIPS; phAIP and phPFG: putative human homologs of AIP and PFG; pMTG: posterior MTG and MTG_t_: sematic tool processing in MTG; LOC: lateral occipital complex; LOC_o_, LOC_a_, LOC_t_ parts of LOC devoted to objects, action observation, and tools.

In the original experiments of Peeters et al. (2009), the left aSMG region was defined as the conjunction of all the interactions for observing the different tool actions and the robot hand actions. This conjunction yielded 75 voxels (red outlines in **Figure 1**) located in the anterior tip of left SMG and centered at Talairach coordinates -60, -21, 31. These voxels likely underestimate the region devoted to tool action observation. Indeed, the subsequent study (Peeters et al., 2013) yielded a similar region of interaction for observation of all three tool actions (rake, pliers, screwdriver) combined, but located just to either side of the original 75 voxels (yellow outlines in **Figure 1**). Furthermore, testing the interaction in individual subjects yielded a moderate dispersion of the local maxima, less so in female than in male subjects. Therefore in **Figure 1** we consider the combination of the three components (red and yellow outlines) showing significant interaction in either experiment (Peeters et al., 2009, 2013) as a more realistic estimate of the left aSMG, but these 180+ voxels most likely still remain an underestimate.

The area is located in the anterior part of the crown of the SMG (**Figure 1**), below SI, posterior to gustatory cortex (BA 43), and dorsal to the parietal opercular areas (Eickhoff et al., 2006). Thus any further extension of aSMG is likely to occur in the dorso-caudal direction. Area aSMG largely overlaps with cytoarchitectonic area PFt (Caspers et al., 2008; Peeters et al., 2013), a remarkable match given all the uncertainties of defining each of these functional and anatomical entities in different groups of subjects by very different means, and ensuring that they are properly registered to one another. Using on a tractography-based parcellation of IPL, Mars et al. (2011) assigned the aSMG region to PFop (Caspers et al., 2008), which they consider distinct from more posterior SMG regions activated during grasping movements. The aSMG region exhibits a considerable anatomical asymmetry with a left hemisphere bias (Van Essen et al., 2012), and the action observation activation is also completely asymmetric, being restricted to the left hemisphere (Peeters et al., 2009). Activation of the aSMG region is also strongly asymmetric in the planning of pantomimes of tool use (Króliczak and Frey, 2009) and its functional connectivity is also asymmetric (Zhang and Li, 2013). This left lateralization only partially reflects the leading role of the right dominant hand in tool manipulation, as these activations have been shown invariant for handedness (Króliczak and Frey, 2009; but see Lewis et al., 2006; Martin et al., 2011). Finally it is worth remembering that deficits in tool handling are a hallmark of apraxia, which is generally associated with left IPL lesions (Goldenberg and Hagmann, 1998). In the opposite hemisphere the symmetrical region is occupied by the higher-order parietal motion area devoted to the extraction of attention-based motion, including long range apparent motion (Claeys et al., 2003).

**FIGURE 2:**
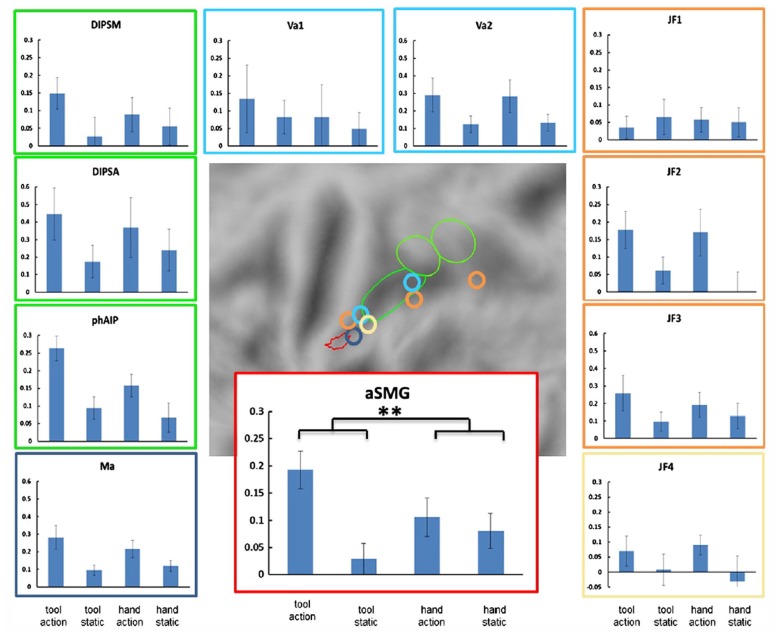
**Specificity of left aSMG amongst parietal regions for tool action observation (from Peeters et al., 2013).** Activity profiles of the 11 ROIs with locations of these ROIs shown in the middle panel. 10 ROIs were defined in left parietal cortex: aSMG, phAIP and DIPSA, DIPSM (Peeters et al., 2009), three tool related ROIs from Johnson-Frey et al. (2005; JF1-3), two from Valyear et al. (2007; Va1-2) and one from Mahon et al. (2007; Ma). The eleventh ROI, located in the right parietal cortex (Johnson-Frey et al., 2005) is drawn on the symmetrical position in the left hemisphere (JF4). The conditions shown in the activity profiles are tool action observation, static tool, hand action observation, and static hand. Vertical bars indicate SEM. The black asterisks indicate the only ROI in which the interaction between tool and action was significant (*p* < 0.05 corrected for 11 comparisons): aSMG.

The aSMG region was originally discovered using movies showing actions performed with tools and compared with similar actions performed with the hand, a paradigm which at that time had never been tested in fMRI. The aSMG overlaps partially with the left lateralized tAIPS region (Mruczek et al., 2013) defined by the subtraction viewing static tools vs. viewing static animals. A long list of imaging studies clearly indicate that the aSMG area is also activated by the sounds produced by tools when used (Lewis et al., 2005), as well as by executing tool actions, imaging or pantomiming tool use or making decisions about tool use (Binkofski et al., 1998; Chao and Martin, 2000; Moll et al., 2000; Okada et al., 2000; Inoue et al., 2001; Rumiati et al., 2004; Bunzeck et al., 2005; Creem-Regehr and Lee, 2005; Johnson-Frey et al., 2005; Valyear et al., 2007; Jacobs et al., 2009; Króliczak and Frey, 2009; Gallivan et al., 2013). The activation during both observation and execution of tool actions suggests that the aSMG may house mirror neurons (Rizzolatti and Craighero, 2004) for tool actions. Finally, the rostral part of SMG is activated when individuals make prehistoric tools or observe their production (Stout and Chaminade, 2007; Stout et al., 2008, 2011). Most of these imaging studies report the activation of several parietal activation sites, in addition to the aSMG. With respect to activation by tool action observation, Peeters et al. (2013) were able to show the uniqueness of aSMG (**Figure 2**). This region was the only parietal region of interest (ROI) out of 11 exhibiting a significant interaction between the factors *action* and *tool*. Indeed the activity profile indicates that, as in the original Peeters et al. (2009) study, the differential activation for observing tool actions significantly exceeded the differential activation for observing biological actions.

Peeters et al. (2013) showed that the left aSMG was activated as much by the observation of rake actions as by the observation of a human hand dragging an object like a rake. Although the activation of the aSMG by rake action observation was relatively weak, the same observation was made with two different groups of subjects. This finding suggests that what is critical is the observation of tool actions are the kinematics of the action, which are very different for tools and biological effectors. It further suggests an important distinction between the activation of the putative human homolog of anterior intraparietal (phAIP) and aSMG during observation of tool actions. Area phAIP is activated by observing the tool being grasped (Jacobs et al., 2009), just as for any other object, explaining why phAIP is also activated when observing biological manipulative actions. On the other hand, aSMG is activated by observing the tool being moved to achieve the goal (picking up or dragging the object toward the actor). One may therefore extrapolate and suggest that during execution phAIP and aSMG play similar roles, with phAIP planning the grasp of the tool and aSMG planning the movement of the tool to obtain the goal.

In conclusion functional imaging provides compelling evidence that the aSMG region, localized in the anterior tip of left IPL is specialized for processing of diverse aspects of tool use, using kinematics as the main visual feature of tool action.

## NEURAL NETWORKS FOR TOOL ACTION EXECUTION AND OBSERVATION

In this section we propose schemes for the afferent and efferent connections of phAIP (bilaterally, but with left dominance if right-handed subject) and left aSMG, operating in parallel during action execution and observation. These schemes will combine knowledge of connections of monkey cortical regions for which homology is known or plausible, as well as some human connectivity data, which by nature are indirect and must be considered with circumspection.

### HAND AND TOOL ACTION EXECUTION

**FIGURE 3:**
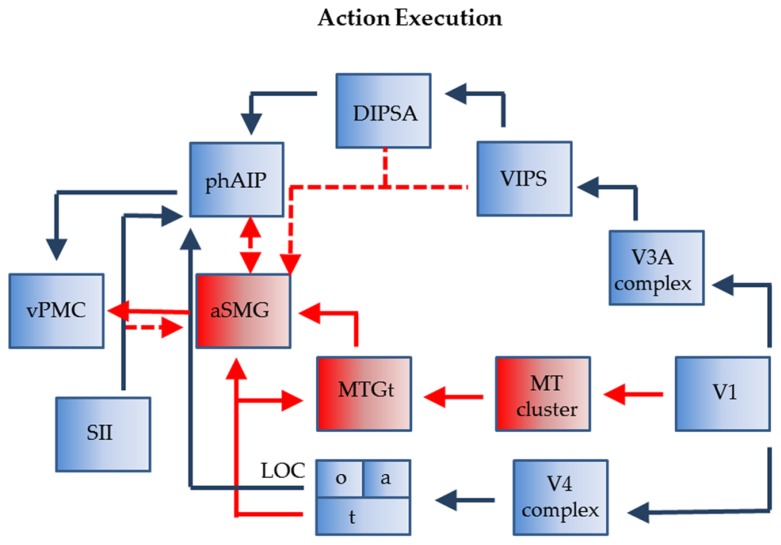
**Connections of phAIP and aSMG during execution of actions: biological actions (blue) tool actions (blue + red).** Dashed lines: postulated connections; Abbreviations see **Figure 1**.

The connections of phAIP and aSMG operating when agents plan for the use of a tool are shown in **Figure 3**. Left phAIP and left aSMG act in parallel, possibly exchanging lateral connections, and send converging signals to vPMC. The parallel nature of this operation needs qualification as only phAIP is active in planning hand actions, while both phAIP and aSMG operate during planning of tool actions. Convergence upon vPMC is supported by the evidence that, in the monkey, the same F5 neurons are active during the last phase of the grasping, i.e., the closure of the effector, regardless of whether the action is performed with the hand, with a tool, or even with a tool requiring an opposite biomechanical movement (finger opening rather than closing) to grasp the object (Umiltà et al., 2008). It is noteworthy that the parallel operation of phAIP and aSMG is a further elaboration of the ventro-dorsal stream (Rizzolatti and Matelli, 2003) of the dorsal visual pathway, or its human homolog. This does not imply that planning tool and hand actions are the only function of the ventro-dorsal stream, or that the present scheme is the only possible elaboration of this stream. Our view is related to those of Daprati and Sirigu (2006) and Binkofski and Buxbaum (2013), who both consider a role for the ventro-dorsal stream in tool use, but also stress the role of the dorso-dorsal stream in planning hand actions toward objects.

The monkey AIP is involved in planning grasping and other manipulative action using visual signals indicating the shape and size of objects; more specifically, this region has a visual component, located in the posterior sector and housing neurons selective for 3D shape from stereo (Srivastava et al., 2009), and a motor component, located in the anterior sector and housing visuomotor and motor neurons (Murata et al., 1996, 2000). Like monkey AIP, its human homolog comprises an anterior sector with motor and visuomotor properties, phAIP, and a posterior visual sector, the dorsal IPS anterior (DIPSA) region, probably equivalent to IPS5 (Mruczek et al., 2013), which, most likely, play similar roles in grasping various objects, including tools. In the monkey, AIP receives from caudal IPS (CIP), that itself receives from V3A (Nakamura et al., 2001). In humans, V7 and its twin area V7A (Georgieva et al., 2009; Kolster et al., 2011), likely equivalent to IPS0-1 (Silver et al., 2005), may correspond in the monkey to the CIP1-2 pair (Arcaro et al., 2011; Janssens et al., 2013). V7 overlaps heavily with a motion-sensitive area ventral intraparietal sulcus (VIPS; Sunaert et al., 1999) which is incorporated in the present scheme. In humans, the region corresponding to the monkey’s V3A may have expanded and may include the four areas described in the V3A complex by Georgieva et al. (2009). Hence we indicate in **Figure 3** input to DIPSA from the V3A complex via VIPS. In the monkey AIP also receives input from the lower bank of superior temporal sulcus (STS; Borra et al., 2008) which supposedly provides input concerning object properties. In deference to the homology between the monkey IT and the human lateral occipital complex (LOC; Denys et al., 2004), we show an input from a subregion of LOC (LOC_o_), on the ventral occipito-temporal surface, to phAIP. LOC receives from the V4 complex in the ventral pathway.

With regard to aSMG, it has been demonstrated that this region is connected with vPMC and posterior middle temporal gyrus (pMTG; Ramayya et al., 2010). This latter region is presumed to represent the tool use associated motion (MTG_t_; Chao et al., 1999; Devlin et al., 2002; Johnson-Frey et al., 2005; Mruczek et al., 2013), corresponding to the “law” of the tool, i.e., defining its nature (the target-movement mapping of the tool; Massen and Prinz, 2007). It probably provides the main input to aSMG for its planning function. MTG_t_ itself processes dynamic input (Beauchamp et al., 2002), and is located close to the MT cluster (Kolster et al., 2010), from which it may receive input. MTG_t_ also receives input from the fusiform gyrus representing semantic knowledge about tool properties (LOC_t_), a fusiform region also projecting directly to aSMG (Mahon et al., 2007). Which exact part of LOC devoted to tools requires further exploration as more lateral regions have also been implicated (Peelen et al., 2013; but see Mruczek et al., 2013). The connection between LOC_t_ and aSMG, thus linking ventral and dorsal streams, is similar in nature to that from LOC_o_ to phAIP. It is unclear to what extent aSMG needs visual input from the ventro-dorsal stream. So far we have assumed that aSMG receives no specific visual input. However, to utilize the tool, it has to be positioned appropriately with respect to the target object (consider a screwdriver and the slot in the screw); in addition once the tool is moving, the target is subject to its influence and will also be moving (in this instance the screw getting deeper). Both aspects, as they relate to the application of the *law* of the tool, may require visual input for proper assessment. Possible sources of such information may be the dorsal IPS medial (DIPSM) or VIPS (**Figure 3**). In this way the aSMG region subserves the tool-object relationship, while phAIP takes care of the tool-actor relationship (Osiurak et al., 2009). Finally, aSMG is likely to receive tactile input indicating whether and how the tool is being held by the hand. These inputs may originate in neighboring SII and reach aSMG directly or indirectly via phAIP. Indeed, the anterior part of monkey AIP receives input from SII (Borra et al., 2008).

### HAND TOOL ACTION OBSERVATION

**FIGURE 4:**
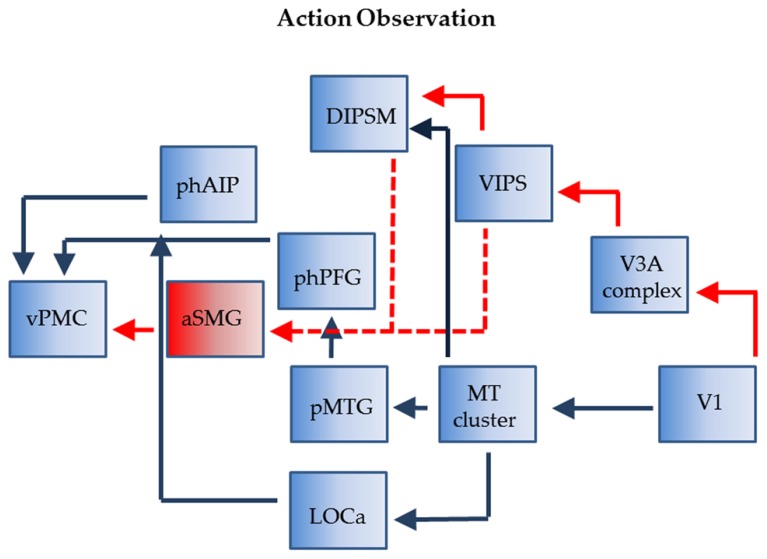
**Connections of phAIP and aSMG during observations of actions: biological actions (blue) tool actions (red + blue).** Dashed lines: postulated connections; Abbreviations see **Figure 1**.

During the observation of tool actions phAIP and aSMG again operate in parallel and again their outputs converge onto vPMC (**Figure 4**). Although the exact homology of vPMC is under investigation (Orban et al., 2012) it likely includes the homolog of monkey F5c, an area which contains the mirror neurons. This convergence is supported by the evidences that, in trained monkeys, the observation of tool use triggers activity in hand grasping mirror neurons (Rochat et al., 2010), even if the firing rate of these neurons was higher during hand grasping observation. Furthermore, the lack of interaction between *tool* and *action* in the studies of Peeters et al. (2009, 2013), confirmed that vPMC is as differentially active for observing biological actions as for tool actions. The most plausible reason of this convergence in vPMC is that, during both hand and tool action observation, the motor cortical areas respond in relation to the goal of the action, which is the same in both types of action, rather than to the movement executed to accomplish the goal (Järveläinen et al., 2004; Cattaneo et al., 2009).

In the monkey the visual signals concerned with grasping observation transit through two parietal stations, AIP and PFG (Nelissen et al., 2011). The homolog of PFG (tentatively labeled phPFG) is still unknown but some recent data (Ferri et al., 2013) suggest that it is located ventrally and slightly caudally from phAIP. The two parietal regions, AIP and PFG, receive input from monkey STS, more precisely with middle superior temporal pole (STPm) in the upper bank feeding into PFG and a region in the rostral lower bank of STS located near that providing object property information, projecting to AIP. We assume the same holds in humans and given the assumed homology (Jastorff and Orban, 2009; Jastorff et al., 2012) of STPm with pMTG and of the rostral lower bank of STS with lateral part of LOC (LOC_a_; **Figure 1**) we indicate such connections in **Figure 4**. Both regions likely receive input indirectly from the MT cluster, which is very similar in monkeys and humans (Kolster et al., 2009, 2010). In both species this cluster includes four areas sharing central representations in the center of the cluster.

While it is relatively clear how visual information about observed biological actions reach phAIP, it is less clear how visual signals related to observed tool actions reach aSMG. The most likely origin is from posterior parietal cortex (PPC). One possibility is through DIPSM, another motion-sensitive region which is the homolog of anterior LIP (Durand et al., 2009), also a motion-sensitive region in monkeys (Freedman and Assad, 2006; Orban et al., 2006). LIP in the monkey receives from the MT cluster, and DIPSM likely does the same. The alternative is through VIPS, which we believe receives input from human V3A, which is also motion-sensitive (Tootell et al., 1997), unlike it monkey counterpart (Vanduffel et al., 2001). These routes over the PPC may explain why more parietal regions are activated by motion stimuli in humans than in monkeys, whether by 3DSFM motion (Vanduffel et al., 2002) or translation (Orban et al., 2006).

## RELATIONSHIP WITH NEUROPSYCHOLOGICAL AND COGNITIVE STUDIES

### THE RELATIONSHIP BETWEEN TOOL ACTIONS AND AFFORDANCES

A difficulty in studying brain mechanisms involved in tool use arises in attempting to formalize for scientific purposes the folk category of tool. In fact, each definition of a tool, attempting to distinguish between tool use and other behaviors, has proven elusive and often led to paradoxical conclusions. Hence, many investigations into what would correspond to tool use have generally concluded that this behavioral category is arbitrarily drawn, and that any definition of tool use is one of convenience rather than psychological (Beck, 1980; Preston, 1998; Osiurak et al., 2010). Part of the problem arises from studies in animals, for which the distinction between tool use and other purposeful behavior using natural materials such as branches, e.g., building a nest, is less clear. These difficulties also reflect the fact that humans, who use tools proficiently, use many different types of artifacts with diverse goals, i.e., extending various natural actions, e.g., using a car or a rifle. The solution might be that a tool is any artifact extending the class of manipulative actions, with the understanding that tools will generally be man-made artifacts for humans, but not for animals. Can a spoon or a knife be considered a tool? The answer depends very much on the goal (Osiurak et al., 2010): if they are used to eat, rather than to cook or prepare food, they may not, strictly speaking, belong to the category of tools, if we define tools as artificial implements intended to extend human manipulative capability. The same applies to a toothbrush (Sunderland et al., 2013), which extends the interaction capabilities with the own body and not with external objects. Recent evidence (Ferri et al., 2013) indicates that viewing actions directed to the own body activates different parietal regions from viewing manipulation. While the boundaries of what counts as a tool are still fuzzy, a number of manipulable objects can clearly not be considered tools and we would emphasize the need for greater care in the selection of the appropriate stimuli for studying tool use. In contrast, some studies make the opposite error, considering typical tools such as hammers or saws to be objects (Kalénine et al., 2010), and erroneously labeling the aSMG as a region involved in action understanding in general.

A different sort of inappropriate stimuli concerns the use of static images instead of movies. The dynamical aspects, such as kinematics, are intrinsic characteristics of the tool actions detected by aSMG and for this reason static photographs of a gesture (Osiurak et al., 2009; Bach et al., 2010) are not optimal stimuli for studying this region. Indeed, we used static gestures as control conditions. Finally, tool use implies a goal to be accomplished and for this reason one has to take care how pantomime tests are performed: if they simply imitate tool manipulation without addressing any goal (Osiurak et al., 2009), they may not actually fit the definition of a tool action which, by definition, has a goal. In fact, while little is known about the selectivity of aSMG for the goal of the action, it is well known that other regions involved in tool use show dramatic changes in activity between observing an object-directed tool action and observing the same actions devoid of any goal, i.e., when the object on which the tool operates is lacking (Järveläinen et al., 2004; Cattaneo et al., 2009).

The distinction between planning object-directed actions using the natural effectors and planning actions directed to the same object but using tools is consistent with the view (Osiurak et al., 2009) that affordances apply to tools only insofar as they are objects that can be grasped. Considering only tool affordances to gain insight into the use of tools may be counterproductive, as these characteristics fail to consider the relationship between the tool and the object, or target, of the tool action, e.g., the nail when using a hammer, or the screw when using a screwdriver (Osiurak et al., 2009). Affordances are egocentric, while we need an allocentric framework to plan tool use, particularly that of novel tools (Osiurak, 2013). Hence, it is highly unlikely that only the affordance of tools, even very familiar ones, can explain the repetition suppression effects observed by Valyear et al. (2012) during tool observation. Indeed the suppression effects were induced by repeated visual presentation of a tool, and not by repeating the tool action. Hence these effects are unlikely to track the existence of specialized neuronal mechanisms for the use of these familiar tools, the more that repetition suppression is known to overestimate selectivity (Sawamura et al., 2006).

**FIGURE 5:**
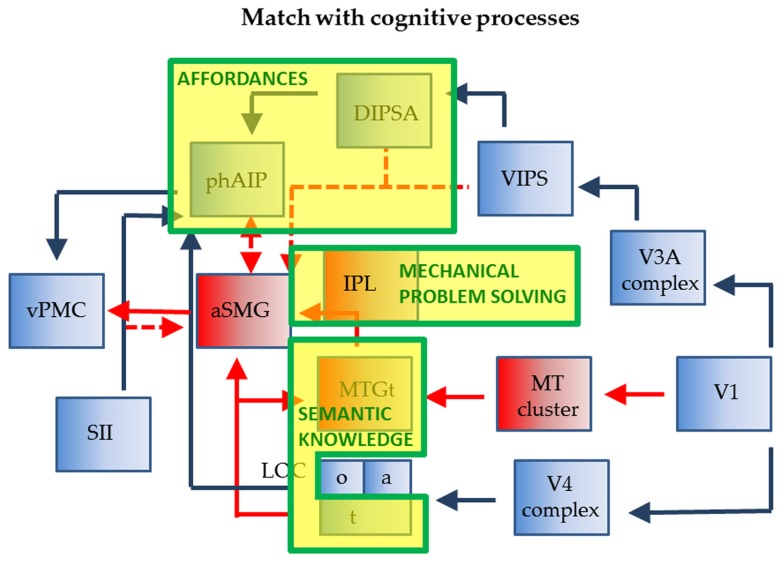
**Relationship of wiring diagram of phAIP and aSMG (execution) to cognitive processes indicated as yellow boxes with green outlines: affordances, technical reasoning, and sematic knowledge**.

The relationship between tool use and affordances can be clarified using **Figure 3**. It indicates that the tool action planning network is more complex, as two parietal regions are involved in planning tool use: phAIP to grasp the tool and aSMG to move it according to its nature. Considering the final goal to be reached by the tool action, affordances apply only to the phAIP component of the planning, which is indeed the component common to both objects and tools (**Figure 5**). However, this does not exclude the possibility that aSMG send some biasing signal to phAIP to favor the selection of the affordance that best suits the proper use of the tool. The phAIP affordance component uses the visual analysis of size and shape to plan the appropriate grip aperture, a function commonly associated with the canonical neurons described in monkey AIP and F5, i.e., with the ventro-dorsal stream, unlike what is proposed by Osiurak (2013). That visual analysis generally yields several affordances, one of which has to be selected according to the goal of the action and the agent’s intentions, a function in which prefrontal cortex (PFC) afferents to AIP may play a role. As far as tool grasping is concerned, aSMG is the primary candidate region for signaling the most appropriate affordance to phAIP. This view is supported by the finding that patients with ideomotor apraxia perform more poorly in grasping tools correctly than in grasping abstract objects (Sunderland et al., 2013), thus suggesting that the selection of the *more appropriate* grip of a tool depends on both phAIP coding multiple affordances and aSMG providing knowledge of the *law* of the tool.

### APRAXIA AND MECHANICAL PROBLEM SOLVING IN TOOL USE

The left lateralization of the planning of tool use is consistent the observation made repeatedly, that tool use is deficient in apraxia due to left parietal lesions (Goldenberg and Hagmann, 1998). The difficulty has been in appreciating that the various symptoms included in apraxia, typically deficits in imitation of meaningless gestures and in tool use, need not to be necessarily related. Attempts have been made to link these two deficits and find a common underlying factor such as the analysis of spatial relationships (Goldenberg, 2009), but recent studies have challenged this view showing that apraxic patients perform more poorly in tool-related actions than in hand actions, even if the demands of these tasks on postural or spatial representation are identical (Sunderland et al., 2013). The common association of deficits in reproducing meaningless gestures and tool use may simply indicate that the neural mechanisms of these two activities while distinct, occupy neighboring IPL regions. This juxtaposition could explain their common involvement in lesions which are generally due to stroke and typically involve large areas of cortex. More fundamental and productive was the realization that the use of familiar tools which is generally impaired in apraxia may in fact be dependent on two distinct mechanisms (Goldenberg and Spatt, 2009). One concerns semantic knowledge of the conventional use of familiar tools and the other the inference of function from structure (Goldenberg and Hagmann, 1998; Daprati and Sirigu, 2006), also referred to as mechanical problem solving (Goldenberg and Spatt, 2009) or technical reasoning (Osiurak et al., 2010).

The present review provides clear indications regarding the neural pathways that may underlie semantic knowledge of the conventional use of familiar tools, a circuit which relies on the LOC_t_ and MTG_t_, two areas providing input to the aSMG. Thus aSMG indeed constitutes the entry point of semantic information into the dorsal pathway, extending what was already known about AIP/phAIP. In contrast, the present review has provided no additional information with regard to the other component of tools use, mechanical problem solving, which can be applied to new tools as well as to non-conventional uses of familiar tools. This type of reasoning refers to the ability to contemplate the abstract principles and mechanics involved in tool use, and is based on mental simulations (Hegarty, 2004) relying on high-level allocentric spatial representations (Osiurak, 2013) and analog processes involved in rule-based reasoning. Importantly, technical reasoning does not require semantic knowledge (Osiurak et al., 2010). Indeed, a decision that the tip of a screwdriver is appropriate for the groove in a particular screw is relatively independent of our semantic knowledge about screwdrivers, in that the relevant information for turning screws is the fit of the tip of one object, whatever its’ nature (a screwdriver, a knife, or a coin), into the slot of the other object. That aspect suggests some interesting parallelism with affordances, which also do not require explicit knowledge of object identity, the main difference being that during tool use the hand-object relationship, typical of affordances, is replaced by the relationship between the tool and its receiver object (Osiurak et al., 2010).

There is now mounting evidence from apraxia studies that mechanical problem solving/technical reasoning must be localized in the left IPL (Goldenberg, 2009; Osiurak et al., 2010). One possibility is that the region located caudal to aSMG, between aSMG and phAIP, extending toward the angular gyrus, is involved in this function (? in **Figure 1**). A possible indication is provided by the original Peeters et al. (2009). In this study, one of the tools, the screwdriver, was used in an unconventional way, to pick up an object. The activation for observing this action extended much further posterior that that evoked by observing the rake or the pliers being used conventionally (compare Figure 2B with Figures 2C,D in Peeters et al., 2009). The mechanical problem-solving function was probably also active in the subjects of Stout et al. (2011), who observed Acheulean tool making, compared to Oldowan tool making. Again, the activation common to all type of subjects (novices, trained, experts) extended further caudally (LM -50, -36, 42) compared to the aSMG as defined in Peeters et al. (2009, 2013). Thus the aSMG (**Figure 5**) would be the entry-point for not only semantic information into the dorsal stream, but also for the output of technical reasoning, hence the locus of the dialectic as described by Osiurak et al. (2010).

## THE ORIGIN OF aSMG DURING THE EVOLUTION

As mentioned earlier the chimpanzee tool use differs fundamentally from that of humans (Osiurak et al., 2010; Vaesen, 2012), depending primarily on a modification of the biological grasping circuit centered on AIP. This is supported by recent evidence that chimpanzees differ as much from humans as macaques do with respect to action observation (Hecht et al., 2013). Hence the question arises as to when tool use based on left aSMG arose during evolution. The first ancestors equipped with this new neural apparatus were most likely Homo Habilis, associated with the Oldowan industrial complex or Homo Erectus, associated with the Acheulean industrial complex. The former dates back 2.5 million years, the second to 1.7 million years before present (Asfaw et al., 1992; Susman, 1994; Roche et al., 1999). Either choice would place the development of tool use, between the emergence of the two other main human traits, bipedalism and language. Bipedalism can be traced back to the Australopithecus, a species much older than Homo Ergaster or Erectus, that emerged about 4 million years ago (Wood and Baker, 2011). However, Homo erectus was probably the first fully fledged biped (Ruff, 2009). Language on the other hand is a much more recent acquisition and may be as recent as 600 thousand years, or less, following the emergence of the premodern homo (Coqueugniot et al., 2004), who was a maker of composite tools.

This timing suggests that these three developments may to some degree be interdependent. Indeed bipedalism frees the hands for manipulative purposes, and must have been an important step toward tool use, insofar as tools are obviously manipulated by the hand. One possibility is that a region such as AIP was duplicated by a prolongation of the cell cycle and this region gradually came to be controlled by afferents carrying tool use related signals. On the other hand, if tool use did indeed precede the emergence of language, it may help understand the typical left lateralization of language. Indeed tool use may in fact have triggered the development of technical reasoning in left IPL, which in turn may have favored a development of language in the left hemisphere. The link between the emergence of tool making and language during evolution has been postulated previously (Stout et al., 2008; Stout and Chaminade, 2012). This view receives support from the involvement of certain IPL regions neighboring those involved in technical reasoning in literacy (Carreiras et al., 2009), the understanding of words, and probably also the planning of speech (Wernicke area or Spt, Hickok and Poeppel, 2007). An evolutionary link is also supported by the modest asymmetry favoring the left hemisphere which has been observed in non-human primates (Joly et al., 2012). It has been argued that the left asymmetry of language is much clearer for execution than for understanding (Hickok and Poeppel, 2007). Execution includes the planning of speech and thus a parietal component, and we may consider Wernicke’s area as a region of sensori-motor transformation (Cogan et al., 2014), just as most other PPC regions.

## CONCLUSION

A large body of imaging studies implicates the left aSMG region as an area involved in the execution and observation of tool actions. The present review has attempted to make the implications of these findings explicit, particularly with respect to pathways centered on the anterior parietal sulcus regions: phAIP and aSMG, the latter appearing to be a specialization of phAIP for manipulating complex tools. Switching from hand to tool action requires, besides the visual input regarding features of the target object provided by the IPS to phAIP and aSMG (for hand-object and tool-object interaction, respectively), additional information specific to tool use and presumably converging upon aSMG. These afferents include semantic information, which is particularly relevant when using familiar tools, technical reasoning, more crucial during the use of uncommon or new tools, and somatosensory feedback. It follows that the affordances of a tool cannot, by themselves, account for tool use. Furthermore, postural and intentional constraints also play a role during the planning of tool actions and consequently are probably provided by inputs to aSMG, which were not discussed. This connectivity model is clearly more elaborated than the two substreams of the dorsal visual pathway. At present it displays a partial convergence with recent neuropsychological views in so far as the semantic input required for familiar tool use has been identified with some degree of confidence, while the cortical localization of technical reasoning can only be conjectured. These views also provide new support for the link between tool-making and language emergence during evolution.

## Conflict of Interest Statement

The authors declare that the research was conducted in the absence of any commercial or financial relationships that could be construed as a potential conflict of interest.
